# The endoplasmic reticulum, not the pH gradient, drives calcium refilling of lysosomes

**DOI:** 10.7554/eLife.15887

**Published:** 2016-05-23

**Authors:** Abigail G Garrity, Wuyang Wang, Crystal MD Collier, Sara A Levey, Qiong Gao, Haoxing Xu

**Affiliations:** 1 Neuroscience Program, University of Michigan, Ann Arbor, United States; 2 Department of Molecular, Cellular, and Developmental Biology, University of Michigan, Ann Arbor, United States; Howard Hughes Medical Institute, Boston Children's Hospital, United States

**Keywords:** lysosome, calcium, ER, Mouse

## Abstract

Impaired homeostasis of lysosomal Ca^2+^ causes lysosome dysfunction and lysosomal storage diseases (LSDs), but the mechanisms by which lysosomes acquire and refill Ca^2+^ are not known. We developed a physiological assay to monitor lysosomal Ca^2+^ store refilling using specific activators of lysosomal Ca^2+^ channels to repeatedly induce lysosomal Ca^2+^ release. In contrast to the prevailing view that lysosomal acidification drives Ca^2+^ into the lysosome, inhibiting the V-ATPase H^+^ pump did not prevent Ca^2+^ refilling. Instead, pharmacological depletion or chelation of Endoplasmic Reticulum (ER) Ca^2+^ prevented lysosomal Ca^2+^ stores from refilling. More specifically, antagonists of ER IP3 receptors (IP3Rs) rapidly and completely blocked Ca^2+^ refilling of lysosomes, but not in cells lacking IP3Rs. Furthermore, reducing ER Ca^2+^ or blocking IP3Rs caused a dramatic LSD-like lysosome storage phenotype. By closely apposing each other, the ER may serve as a direct and primary source of Ca^2+^for the lysosome.

**DOI:** http://dx.doi.org/10.7554/eLife.15887.001

## Introduction

A vacuolar-type H^+^-ATPase (V-ATPase) on the membrane of the lysosome maintains the acidic lumen (pH_Ly_ ~ 4.6), and improper acidification of lysosomes may lead to lysosomal storage diseases (LSDs) (Mindell, 2012). Like the Endoplasmic Reticulum (ER) (Clapham, 2007; Berridge, 2012), lysosomes are also intracellular Ca^2+^ stores with free [Ca^2+^]_Ly_ ~0.4–0.6 mM (Christensen et al., 2002; Lloyd-Evans et al., 2008), which is 3–4 orders of magnitude higher than the cytosolic [Ca^2+^] (~100 nM). A reduction in [Ca^2+^]_Ly_ is believed to be the primary pathogenic cause for some LSDs and common neurodegenerative diseases (Lloyd-Evans et al., 2008; Coen et al., 2012). Using the fast Ca^2+^ chelator BAPTA, Ca^2+^ release from the lysosome has been shown to be required for late endosome-lysosome fusion (Pryor et al., 2000), lysosomal exocytosis, phagocytosis, membrane repair, and signal transduction (Reddy et al., 2001; Lewis, 2007; Kinnear et al., 2004). Consistently, the principal Ca^2+^ channel in the lysosome, Mucolipin TRP channel 1 (TRPML1 or ML1), as well as lysosomal Ca^2+^ sensors such as the C2 domain–containing synaptotagmin VII, are also required for these functions (Steen et al., 2007; Lewis, 2007; Kinnear et al., 2004). Whereas human mutations of *TRPML1* cause type IV Mucolipidosis, pathogenic inhibition of ML1 underlies several other LSDs (Shen et al., 2012).

How the 5000-fold Ca^2+^ concentration gradient across the lysosomal membrane is established and maintained is poorly understood. The most well understood Ca^2+^ store in the cell is the ER. Upon store depletion, the luminal sensor protein STIM1 oligomerizes to activate the highly Ca^2+^-selective ORAI/CRAC channels on the plasma membrane, refilling the ER Ca^2+^ store via the SERCA pump (Clapham, 2007; Lewis, 2007; Berridge, 2012). However, depletion of lysosomal Ca^2+^ stores does not induce extracellular Ca^2+^ entry (Haller et al., 1996b). The endocytic pathway may theoretically deliver extracellular Ca^2+^ to lysosomes. However, most Ca^2+^ taken up through endocytosis is lost quickly during the initial course of endosomal acidification prior to reaching lysosomes during endosome maturation (Gerasimenko et al., 1998). In various cell types, when the lysosomal pH gradient is dissipated, either by inhibiting the V-ATPase or by alkalizing reagents such as NH_4_Cl, free *luminal* [Ca^2+^]_Ly_ was found to drop drastically (Calcraft et al., 2009; Christensen et al., 2002; Dickson et al., 2012; Lloyd-Evans et al., 2008; Shen et al., 2012), with no or very small concomitant increase in *cytosolic* Ca^2+^ (Christensen et al., 2002; Dickson et al., 2012). These findings have been interpreted to mean that the proton gradient in the lysosome is responsible for actively driving Ca^2+^ into the lysosome via an unidentified H^+^-dependent Ca^2+^ transporter (Morgan et al., 2011). Because these findings are consistent with studies in yeast showing that the Ca^2+^/H^+^ exchangers establish the vacuolar Ca^2+^ gradient (Morgan et al., 2011), this 'pH hypothesis' has been widely accepted (Calcraft et al., 2009; Christensen et al., 2002; Lloyd-Evans et al., 2008; Morgan et al., 2011; Shen et al., 2012). However, large, prolonged manipulations of luminal pH may interfere directly with Ca^2+^ reporters, and secondarily affect many other lysosomal processes, especially lysosome luminal Ca^2+^ buffering (Dickson et al., 2012), lysosome membrane potential, and fusion/fission of endosomes and lysosomes (Mindell, 2012). Therefore, these hypotheses about lysosomal Ca^2+^ refilling and store maintenance remain to be tested under more physiological conditions. Directly measuring lysosomal Ca^2+^ release has been made possible recently by using lysosome-targeted genetically-encoded Ca^2+^ indicators (Shen et al., 2012) (GCaMP3-ML1; see Figure 1—figure supplement 1A), which co-localized well, in healthy cells, with lysosomal associated membrane protein-1 (Lamp1), but not with markers for the ER, mitochondria, or early endosomes (Figure 1—figure supplement 1B).

## Results

### A physiological assay to monitor lysosomal Ca^2+^ refilling

**Figure 1.**
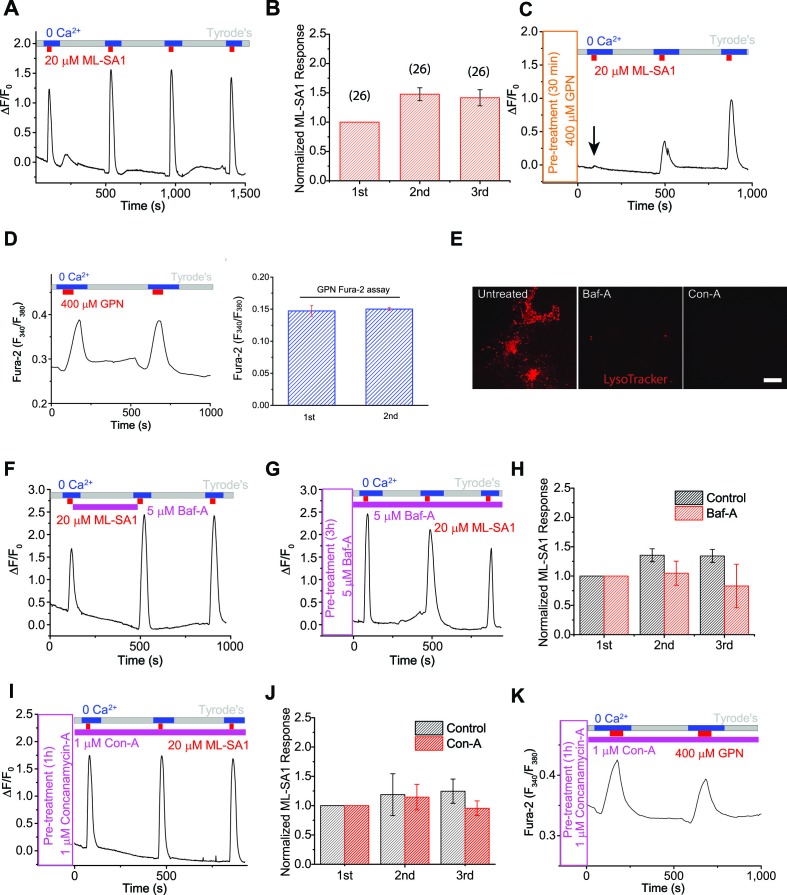
The proton gradient of the lysosome is not required for lysosomal Ca^2+^ store refilling. (**A**) In HEK293 cells stably expressing GCaMP3-ML1 (HEK-GCaMP3-ML1 cells), bath application of the ML1 channel agonist ML-SA1 (20 µM) in a low or 'zero' Ca^2+^ (free [Ca^2+^]<10 nM) external solution induced an increase in GCaMP3 fluorescence (F_470_). After washout for 5 min, repeated applications of ML-SA1 induced responses that were similar to or larger than the first one. Note that because baseline may drift during the entire course of the experiment (up to 20 min), we typically set F_0_ based on the value that is closest to the baseline. (**B**) The average Ca^2+^ responses of three ML-SA1 applications at intervals of 5 min (n=26 coverslips; Figure 1*—*source data 1). (**C**) Pre-treatment with lysosome-disrupting agent GPN for 30 min abolished the response to ML-SA1 in HEK-GCaMP3-ML1 cells. Washout of GPN resulted in a gradual re-appearance of ML-SA1 responses. See quantitation in Figure 1—figure supplement 2K. (**D**) Repeated applications of GPN resulted in Ca^2+^ release that was measured with the Ca^2+^-sensitive dye Fura-2 (F_340_/F_380_) in non-transfected HEK293T cells. (**E**) Application of Bafilomycin-A (Baf-A, 5 µM) and Concanamycin-A (Con-A, 1 µM) quickly (<5 min) abolished LysoTracker staining, an indicator of acidic compartments. (**F**) Acute application of Baf-A (5 µM) for 5 min did not block Ca^2+^ refilling of lysosomes in HEK-GCaMP3-ML1 cells. (**G**) Prolonged pre-treatment (3 hr) with Baf-A did not block Ca^2+^ refilling of lysosomes. (**H**) Quantification of 1^st^ (p value = 0.11), 2^nd^ (p=0.01), and 3^rd^ (p=0.004) ML-SA1 responses upon Baf-A treatment (n=8) compared to control traces (n=6; Figure 1*—*source data 1). (**I**) Prolonged treatment (1 hr) with Con-A did not prevent lysosomes from refilling their Ca^2+^ stores. (**J**) Quantification of 1st (p=0.90), 2nd (p=0.33), and 3rd (p=0.66) ML-SA1 responses with Con-A pre-treatment (n=3; Figure 1*—*source data 1). (**K**) Con-A did not reveal differences in Ca^2+^ refilling responses to repeated applications of GPN in untransfected HEK293T cells. Panels **A**, **C**, **D**, **F**, **G**, **I**, and **K** are the average of 30–40 cells from one representative coverslip/experiment. The data in panels **B**, **H**, and **J** represent mean ± SEM from at least three independent experiments. **DOI:** http://dx.doi.org/10.7554/eLife.15887.002 10.7554/eLife.15887.003Figure 1—source data 1.Source data of Figure 1B, H, J: The average Ca^2+^ responses to ML-SA1 applications under control (**B**), Baf-A1 treatment (**H**), and Con-A treatment (**J**).**DOI:** http://dx.doi.org/10.7554/eLife.15887.003

Monitoring lysosomal Ca^2+^ store refilling requires direct activation of lysosomal Ca^2+^ channels with specific agonists to repeatedly induce Ca^2+^ release. NAADP, the only known endogenous Ca^2+^-mobilizing messenger that has been suggested to be lysosome-specific, was not useful due to its membrane impermeability and strong desensitization (Morgan et al., 2011). Using the specific, membrane–permeable synthetic agonists that we recently identified for lysosomal TRPML1 channels (ML-SA1) (Shen et al., 2012), we developed a lysosomal Ca^2+^ refilling assay as shown in Figure 1A. In HEK293 cell lines stably-expressing GCaMP3-ML1 (HEK-GCaMP3-ML1 cells), bath application of ML-SA1 (30s) in a 'zero' (low; free [Ca^2+^] <10 nM) Ca^2+^ external solution produced robust lysosomal Ca^2+^ release measured by GCaMP3 fluorescence (△F/F_0_ >0.5; Figure 1A,B, Figure 1—figure supplement 1, Figure 1—figure supplement 2A). The membrane-permeable form of the fast Ca^2+^ chelator BAPTA (BAPTA-AM) completely blocked the ML-SA1 response (Figure 1—figure supplement 1D), supporting its Ca^2+^ specificity. Importantly, GCaMP3-ML1-tagged lysosomes co-localized well with LysoTracker, indicating that the pH of these lysosomes was not different from lysosomes without GCaMP3-ML1 (Figure 1—figure supplement 1E).

**Figure 1—figure supplement 1.**
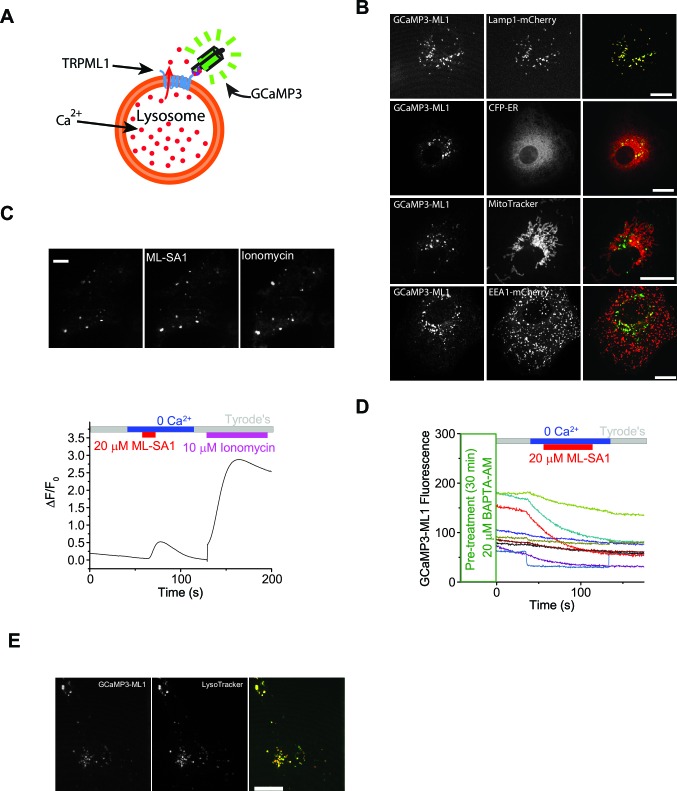
A lysosome-targeted genetically-encoded Ca^2+^ indicator to measure lysosomal Ca^2+^ release, store depletion, and refilling. (**A**) Detection of lysosomal Ca^2+^ release by a genetically-encoded Ca^2+^ indicator (GCaMP3) fused directly to the N-terminus of ML1 (GCaMP3-ML1). (**B**) Co-localization analyses between GCaMP3-ML1 and various organellar markers, including Lamp1-mCherry, CFP-ER, Mito-tracker, and EEA1-mCherry. Scale bars = 5 μm. (**C**) In an HEK-GCaMP3-ML1 cell, both ML-SA1 and subsequent ionomycin induced GCaMP3 fluorescence increases in lysosomes shown by both fluorescence imaging and Ca^2+^ imaging. (**D**) BAPTA-AM pre-treatment abolished ML-SA1-induced responses in HEK-GCaMP3-ML1 cells. Panel C shows the average response of 30–40 cells from one representative experiment. (**E**) Cos7 cells transfected with GCaMP3-ML1 show strong co-localization with LysoTracker, which is highly selective for acidic organelles. **DOI:** http://dx.doi.org/10.7554/eLife.15887.004

**Figure 1—figure supplement 2.**
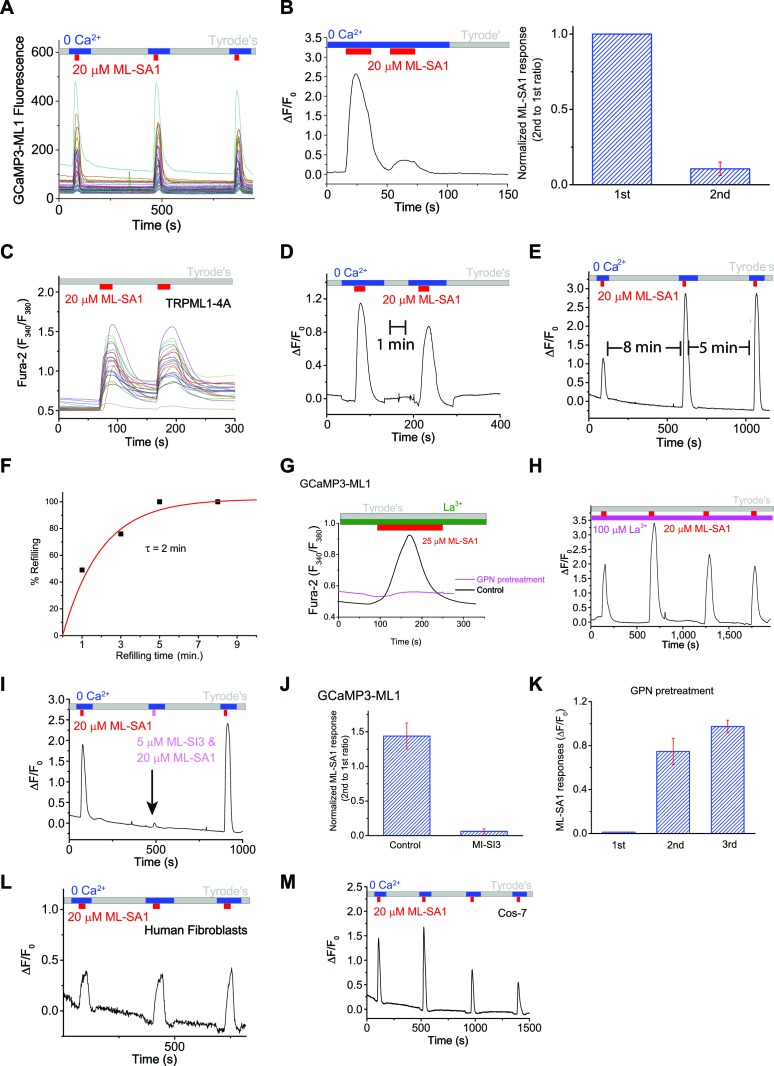
An assay to monitor lysosomal Ca^2+^ store depletion and refilling. (**A**) Raw traces of ML-SA1-induced GCaMP3 Ca^2+^ responses of individual HEK-GCaMP3-ML1 cells on one coverslip. (**B**) Immediate re-application of ML-SA1 showed a nearly-abolished lysosomal Ca^2+^ release. (**C**) In HEK293T cells transfected with surface-expressed TRPML1-4A channels, repeated applications of ML-SA1 induced comparable responses. (**D**) After 1 min refilling time, application of ML-SA1 in HEK-GCaMP3-ML1 cells induced responses that were smaller than the first one. (**E**) The amount of Ca^2+^ released after 8 min of washout and refilling was similar to the amount released after 5 min. (**F**) Time-dependence of lysosomal Ca^2+^ store refilling. (**G**) In the presence of La^3+^ (100 μM), a membrane-impermeable TRPML blocker, GPN pretreatment abolished ML-SA1-induced responses in HEK-GCaMP3-ML1 cells. (**H**) Lysosomal Ca^2+^ refilling in the presence of La^3+^. (**I**) ML-SI3 (5 μM) reversibly inhibited ML-SA1-induced Ca^2+^ responses in HEK-GCaMP3-ML1 cells. (**J**) Normalized ML-SA1 responses with and without co-application of MI-SI3. (**K**) Quantification of ML-SA1 responses shown in Figure 1C. (**L**) Lysosomal Ca^2+^ refilling in human fibroblasts transfected with GCaMP3-ML1. (**M**) Lysosomal Ca^2+^ refilling in Cos-7 cells transfected with GCaMP3-ML1. Panel **B**, **D**, **E**, **G**, **H**, **I**, **L** and M show the average response of 30–40 cells from one representative experiment out of at least independent repeats. **DOI:** http://dx.doi.org/10.7554/eLife.15887.005

**Figure 1—figure supplement 3.**
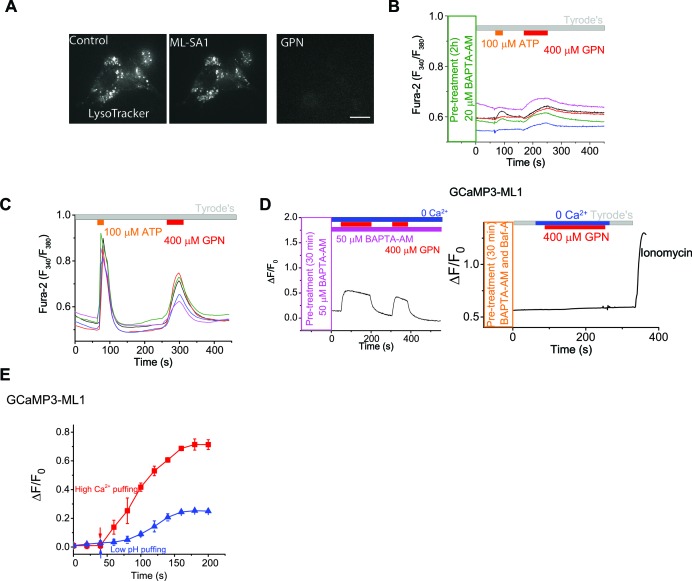
GPN and ML-SA1 have different effects on lysosome pH and GCaMP3 fluorescence. (**A**) LysoTracker staining was not affected by ML-SA1 (20 μM), but was abolished by GPN (400 μM). Scale bar = 15 µm. (**B**) Pretreatment of a membrane-permeable form of Ca^2+^ chelator BAPTA (BAPTA-AM) for 2h abolished Fura-2 response to ATP in HEK293T cells. GPN still induced small increases in the Fura-2 signal in the same cells (also see panel **C**). (**C**) ATP and GPN Fura-2 responses in HEK293T cells. (**D**) GPN (400 μM) induced increases of GCaMP3 fluorescence in HEK-GCaMP3-ML1 cells that were pre-treated and kept in continuous presence of BAPTA-AM, and the increases were abolished by Baf-A1 co-treatment. Note that ionomycin was still able to induce GCaMP3 increases. Panels D show the average response of 30–40 cells from one representative experiment. (**E**) Representative traces showing GCaMP3 sensitivities to patch-electrode-based 'puffing' of low-pH and high-Ca^2+^ solutions to GCaMP3-ML1-expressing vacuoles isolated from HEK-GCaMP3-ML1 cells. **DOI:** http://dx.doi.org/10.7554/eLife.15887.006

**Figure 1—figure supplement 4.**
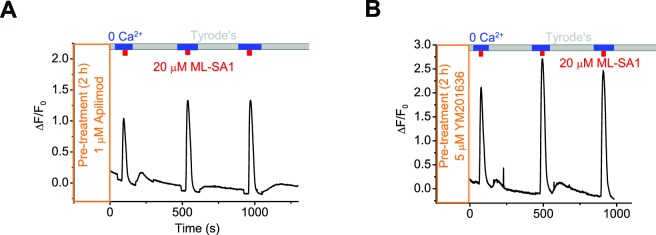
Inhibition of PI(3,5)P_2_ production does not prevent lysosomal Ca^2+^ refilling. Representative Ca^2+^ imaging traces showing lysosomal Ca^2+^ refilling in GCaMP3-ML1 cells pretreated with Apilimod (**A**) or YM201636 (**B**). Panels **A** and **B** show the average response of 30–40 cells from one representative experiment. **DOI:** http://dx.doi.org/10.7554/eLife.15887.007

After release of the initial, 'naïve' Ca^2+^ store upon first application of ML-SA1, lysosomal Ca^2+^ stores are largely depleted, as immediate re-application of ML-SA1 evoked much smaller or no response (Figure 1—figure supplement 2B). The reduction in the second response was unlikely caused by channel desensitization, as surface-expressed TRPML1 mutant (TRPML1-4A [Shen et al., 2012]) showed repeated Ca^2+^ entry in Ca^2+^-containing (2 mM) external solution (Figure 1—figure supplement 2C). Notably, increasing the time interval between consecutive applications quickly and effectively restored the lysosomal ML-SA1 responses; it required 5 min for full restoration/refilling (Figure 1—figure supplement 2D–F). With 5 min of refilling time (chosen for the rest of our experiments), in healthy HEK-GCaMP3-ML1 cells, the second and third ML-SA1 responses are often slightly higher than the first, naïve response (Figure 1A,B).

To ensure the ML-SA1-induced Ca^2+^ responses are exclusively intracellular and lysosomal, all ML-SA1 responses were measured either in the 'zero' Ca^2+^ external solution (Figure 1A) or in the presence of La^3+^ (Figure 1—figure supplement 2G,H), a membrane-impermeable TRPML channel blocker (Dong et al., 2008) that is expected to completely inhibit surface-expressed TRPML1 channels. Ca^2+^ release was completely blocked by the TRPML-specific, synthetic antagonists ML-SI1 or ML-SI3 (Figure 1—figure supplement 2I,J). In addition, pretreatment with the lysosome-disrupting reagent Glycyl-L-phenylalanine 2-naphthylamide (GPN) (Berg et al., 1994) also completely abolished the refilling either in 'zero' Ca^2+^ or in the presence of La^3+^ (Figure 1C; Figure 1—figure supplement 2G), further supporting the lysosome-specificity of the response. The effect of GPN, presumably on so-called 'lysosomal membrane permeabilization', is known to be rapid and reversible (i.e. membrane 'resealing') (Kilpatrick et al., 2013). Consistently, washout of GPN led to gradual recovery of ML-SA1 responses (Figure 1C; Figure 1—figure supplement 2K). Similar Ca^2+^ refilling of lysosomes was also observed in GCaMP3-ML1-transfected human fibroblasts (Figure 1—figure supplement 2L), Cos-7 cells (Figure 1—figure supplement 2M), primary mouse macrophages, mouse myoblasts, and DT40 chicken B cells (Figure 3D–E’). These findings support the idea that these responses are mediated by intracellular Ca^2+^ release from refilled lysosomal stores (also see Figure 1—figure supplement 1C for signals from individual lysosomes). Taken together, these results ensure that lysosomal Ca^2+^ stores can be emptied and refilled repeatedly and consistently in a time-dependent manner.

### Studying lysosomal Ca^2+^ refilling using a lysosome-specific 'membrane-permeabilizer'

GPN is a membrane-permeable di-peptide that is broken down by the lysosome-specific enzyme Cathepsin C. GPN causes permeabilization of lysosome membranes resulting from the accumulation of its breakdown products within lysosomes (Berg et al., 1994). Because it is a lysosome-specific membrane disrupting agent, it is often used to mobilize lysosome-specific Ca^2+^ stores (Jadot et al., 1984; Morgan et al., 2011; Berg et al., 1994; Haller et al., 1996a; Haller et al., 1996b). Using Fura-2 Ca^2+^ imaging in non-transfected HEK293T cells, repeated applications of GPN resulted in a response of similar magnitude to the first, suggestive of Ca^2+^ refilling (Figure 1D). Importantly, in HEK-GCaMP3-ML1 cells, pre-treatment with GPN or BAPTA-AM abolished the initial response to ML-SA1, confirming the GCaMP3-ML1 probe’s lysosome and Ca^2+^ specificity (Figure 1C).

The GPN-mediated 'membrane permeabilizaton' causes the leakage of small solutes including Ca^2+^ and H^+^ into the cytosol (Appelqvist et al., 2012), resulting in changes in the pH (see Figure 1—figure supplement 3A) and [Ca^2+^] in both the lysosome lumen and the peri-lysosomal (juxta-lysosomal) cytosol (Berg et al., 1994; Kilpatrick et al., 2013; Appelqvist et al., 2012). We therefore tested the Ca^2+^-specificity of GPN-induced increases on the Fura-2 and GCaMP3 signals. In cells pretreated with BAPTA-AM, whereas ER-mediated Ca^2+^ responses were abolished, GPN-induced Fura-2 increases were much reduced but not abolished (Figure 1—figure supplement 3B,C). Consistently, in HEK-GCaMP3-ML1 cells pre-treated with BAPTA-AM, GPN still induced a significant increase in GCaMP3 fluorescence. However, in these BAPTA-AM-treated cells, GPN-induced increases in GCaMP3 responses were completely abolished by a pre-treatment of Bafilomycin-A (Baf-A), a specific inhibitor of the V-ATPase (Morgan et al., 2011) (Figure 1—figure supplement 3D). Given that both Ca^2+^ dyes and GFP-based Ca^2+^ indicators are known to be sensitive to other ionic factors, particularly pH (Rudolf et al., 2003), GPN-induced changes in lysosomal and peri-lysosomal pH could directly or indirectly account for the BAPTA-insensitive GCaMP3 and residual Fura-2 signals. Consistent with this prediction, in the vacuoles isolated from HEK-GCaMP3-ML1 cells, GCaMP3 fluorescence was sensitive not only to high Ca^2+^, but also to low pH (Figure 1—figure supplement 3E). Because ratiometric dyes are less susceptible to pH changes (Morgan et al., 2015), in the Fura-2 assay, GPN may induce a large Ca^2+^ signal, but also a pH-mediated contaminating non-Ca^2+^ signal (compare Figure 1—figure supplement 3B with C).

### The pH gradient and V-ATPase are not required for lysosome Ca^2+^ refilling

Next, we investigated the mechanisms underlying Ca^2+^ refilling of lysosomes. Inhibition of endocytosis using dynasore and organelle mobility using cytoskeleton inhibitors such as nocodazole and trichostatin A did not block refilling (data not shown). Furthermore, disruption of Golgi function using Brefeldin-A also had no effect on refilling (Figure 2—figure supplement 1A). Hence, in agreement with previous findings, the secretory and endocytic pathways are not directly involved in Ca^2+^ refilling. PI(3,5)P_2_ is a lysosome-specific phosphoinositide that regulates multiple lysosomal channels and transporters including ML1 (Xu and Ren, 2015). Pharmacologically decreasing PI(3,5)P_2_ levels using two small molecule PIKfyve inhibitors: YM201636 (Jefferies et al., 2008) and Apilimod (Xu et al., 2013) did not prevent lysosomal Ca^2+^ refilling (Figure 1—figure supplement 4A,B).

Previous findings have suggested that the pH gradient in the lysosome may be important to Ca^2+^ refilling (Xu and Ren, 2015; Morgan et al., 2011), however few studies have carefully investigated this possibility. Baf-A and Concanamycin-A (Con-A), inhibitors of the V-ATPase, increase the pH of the lysosome (Morgan et al., 2011), demonstrated by abolishing LysoTracker staining within minutes after application (Figure 1E). Surprisingly, acute application of Baf-A did not affect the response to ML-SA1, and had little effect on refilling (Figure 1F), nor did pretreatment of Baf-A for 1, 3 (Figure 1G,H), or 16 hr. Similarly, pretreatment with Con-A also had no effect on Ca^2+^ refilling of lysosomes (Figure 1I, J, K). These findings suggest that contradictory to previous conclusions, the pH gradient and V-ATPase may not be required for Ca^2+^ refilling, and that an alternative mechanism is responsible for supplying Ca^2+^ to lysosomes.

### The Endoplasmic Reticulum (ER) Ca^2+^ store is required for lysosomal Ca^2+^ refilling

**Figure 2.**
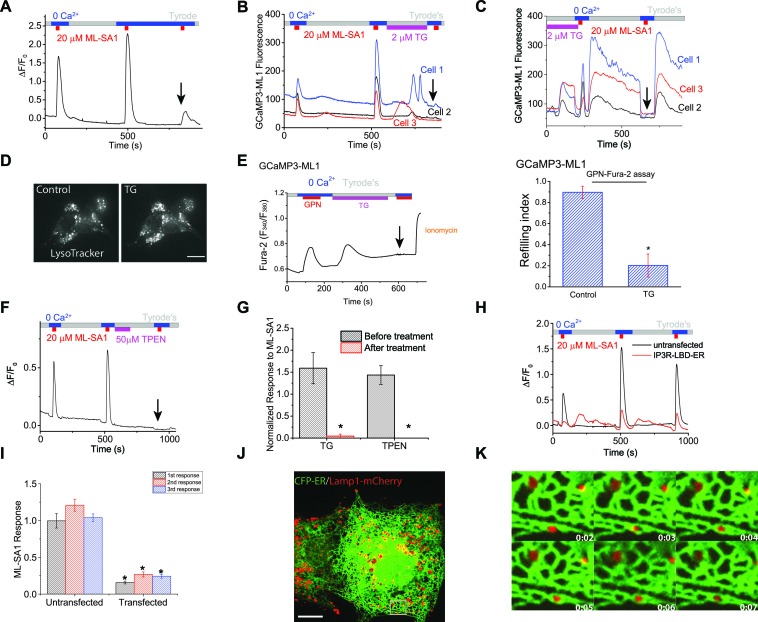
Lysosomal Ca^2+^ refilling is dependent on the endoplasmic reticulum (ER) Ca^2+^. (**A**) Ca^2+^ refilling of lysosomes requires external Ca^2+^. (**B**) Dissipating the ER Ca^2+^ gradient using SERCA pump inhibitor Thapsigargin (TG) blocked lysosomal Ca^2+^ refilling in HEK-GCaMP3-ML1 cells. Three representative cells from among 30–40 cells on one coverslip are shown. Note that Ca^2+^ release from the ER through passive leak revealed after blocking SERCA pumps was readily seen in HEK-GCaMP3-ML1 cells, presumably due to the close proximity between lysosomes and the ER (Kilpatrick et al., 2013). (**C**) The effect of acute application of TG (2 μM) on the naïve ML-SA1 response and lysosomal Ca^2+^ refilling in HEK-GCaMP3-ML1 cells. Application of TG did not affect the naïve, initial response to ML-SA1, but did abolish the refilled response (see arrow). Control naïve response 1.39 ± 0.09 (n=3); Naïve response after TG 1.08 ± 0.07 (n=3); p=0.2024). (**D**) LysoTracker staining was not reduced by TG (2 μM). (**E**) Representative Ca^2+^ imaging trace and statistical data (right panel; Figure 2*—*source data 1) show that TG application reduced the second responses to GPN compared to the control shown in Figure 1D. (**F**) Chelating ER Ca^2+^ using 2-min TPEN treatment blocked Ca^2+^ refilling of lysosomes. (**G**) TG (p=0.008; n=5) and TPEN (p=0.001; n=5) abolished Ca^2+^ refilling of lysosomes (Figure 2*—*source data 1). (**H**) In HEK-GCaMP3-ML1 cells that were transiently transfected with the IP3R-ligand binding domain with ER targeting sequence (IP3R-LBD-ER), which significantly reduces basal [Ca^2+^]_ER_ (see Figure 2—figure supplement 2E), ML-SA1 responses were reduced, compared to untransfected cells on the same coverslip. (**I**) The 1st (p=0.0014), 2nd (p=0.0004), and 3rd responses (p<0.0001) of GCaMP3-ML1 cells transfected with the IP3R-LBD-ER were significantly reduced compared to untransfected cells on the same coverslip (n=5; Figure 2*—*source data 1). (**J**) Lysosomes (labeled with Lamp1-mCherry) interact closely with the ER (labeled with CFP-ER). (**K**) Time lapse zoomed-in images of a selected region from **J** show the dynamics of ER-lysosome association (see an example in the boxed area). Panels **A**, **F**, **H** are the average responses of 30–40 cells from one representative experiment. The data in panel G represent mean ± SEM from five independent experiments. **DOI:** http://dx.doi.org/10.7554/eLife.15887.008 10.7554/eLife.15887.009Figure 2—source data 1.Comparisons of GPN (**E**) and ML-SA1 responses (**G**, **I**) under different pharmacological and genetic manipulations (Figure 2E,G,I).Comparisons of responses to ML-SA1 (**E**) and GPN (**G**) (Figure 2*—*figure supplement 1E,G).**DOI:** http://dx.doi.org/10.7554/eLife.15887.009

Lysosomal Ca^2+^ refilling was drastically reduced upon removal of extracellular Ca^2+^ during refilling time in HEK-GCaMP3-ML1 cells (Figure 2A). However, blocking Ca^2+^ entry using the generic cation channel blocker La^3+^ did not prevent refilling (Figure 2—figure supplement 1B). Because ER stores are passively, although slowly, depleted in 0 Ca^2+^ (Wu et al., 2006) (also see Figure 2—figure supplement 1C), given the demonstrated role of extracellular Ca^2+^ in ER store refilling (Lewis, 2007; Berridge, 2012), we investigated the role of the ER in lysosomal refilling. Thapsigargin (TG), a specific inhibitor of the ER SERCA pump (Thastrup et al., 1990), rapidly and completely abolished Ca^2+^ refilling to lysosomes (Figure 2B,G), but did not affect the first, naïve ML-SA1 response (Figure 2C; second response marked with arrow) or lysosomal pH (Figure 2D). In the GPN & Fura-2 assay that provides a reasonable (but not perfect; see above) measurement of lysosomal Ca^2+^ release independent of ML1, TG application also largely reduced the second GPN response (Figure 1D, 2E), which could be further reduced or abolished by Baf-A pretreatment. These results suggest that TG had no direct effect on the naïve Ca^2+^ store in lysosomes, but specifically and potently affected lysosomal Ca^2+^ refilling. A rapid and complete block of Ca^2+^ refilling was also observed for another SERCA pump inhibitor CPA (Figure 2—figure supplement 1D–G). TG may induce an unfolded protein response (UPR; Matsumoto et al., 2013). However, the UPR inducer Tunicamycin (Oslowski and Urano, 2011) did not affect refilling (Figure 2—figure supplement 1H,I).

**Figure 2—figure supplement 1.**
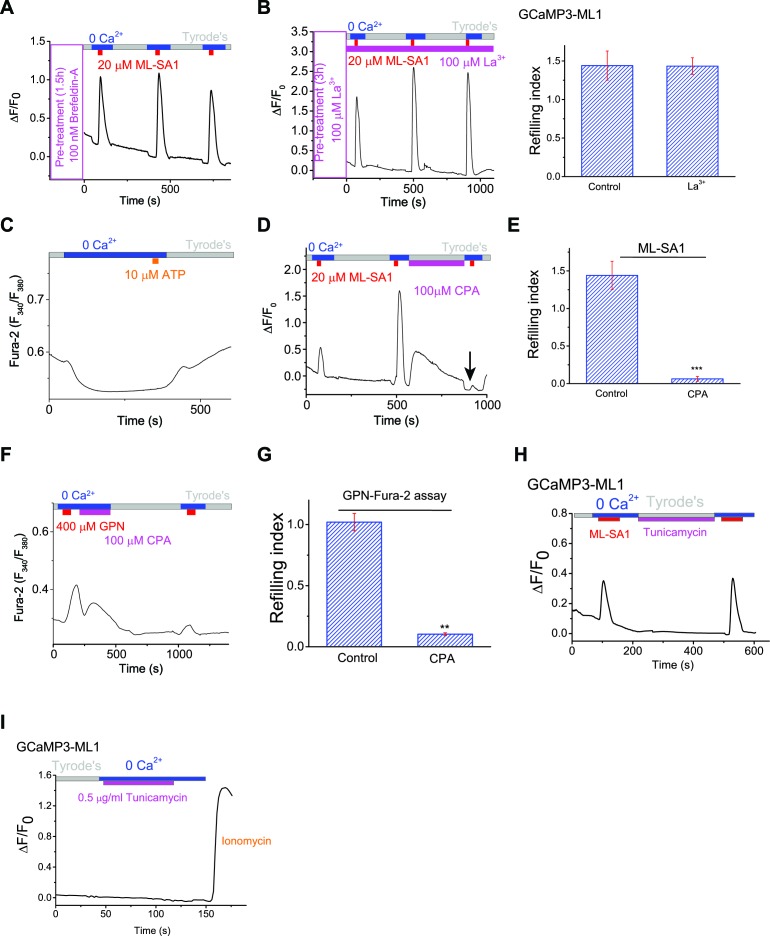
The ER Ca^2+^ store regulates lysosome Ca^2+^ stores. (**A**) The effect of Brefeldin-A (100 nM) pretreatment on lysosomal Ca^2+^ refilling in HEK-GCaMP3-ML1 cells. (**B**) The effect of La^3+^ (100 μM) pre-treatment on lysosomal Ca^2+^ refilling in HEK-GCaMP3-ML1 cells. (**C**) The response to the endogenous P2Y receptor agonist ATP in HEK293T cells loaded with Fura-2 was abolished after perfusing cells with 0 external Ca^2+^ for 5 min. (**D**, **E**) SERCA pump inhibitor cyclopiazonic acid (CPA) (100 μM) blocked Ca^2+^ refilling of lysosomes (Figure 2*—*source data 1). (**F**, **G**) CPA markedly reduced the response to GPN in the Fura-2-loading cells. Note that the remaining response could be due to GPN-induced lysosomal H^+^ release, which resulted in a change of pre-lysosomal pH that may in turn caused a Fura-2 signal (Figure 2*—*source data 1). (**H**) Tunicamycin (0.1 μg/ml) application did not affect the second responses to ML-SA1 in GCaMP3-ML1 cells. (**I**) Tunicamycin (0.5 μg/ml) did not induce lysosomal Ca^2+^ releases. Panels **A**–**I** and **K** show the average response of 30–40 cells from one representative experiment. **DOI:** http://dx.doi.org/10.7554/eLife.15887.010

**Figure 2—figure supplement 2.**
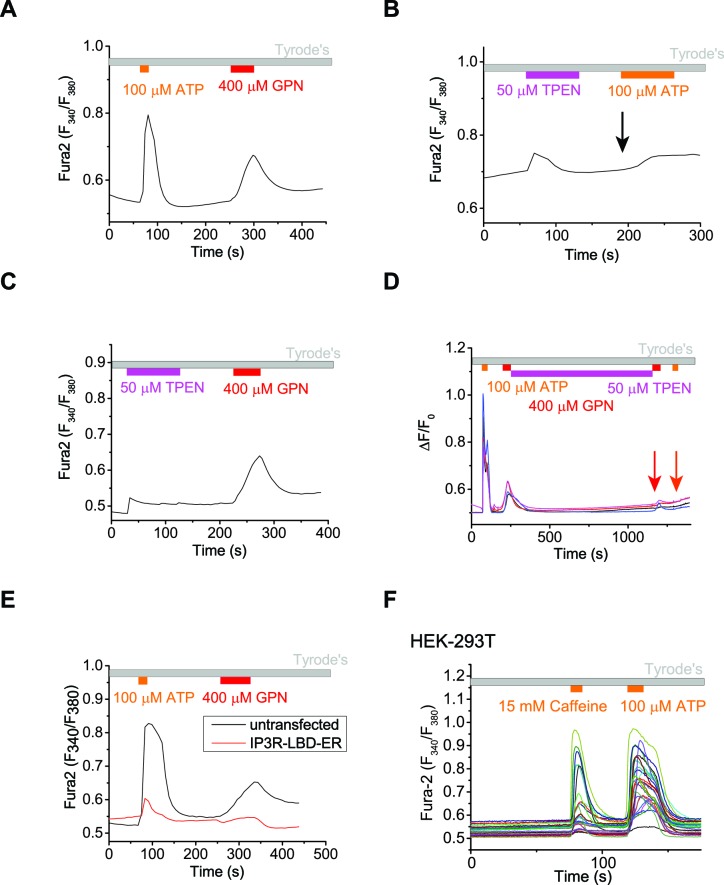
The ER Ca^2+^ store regulates lysosome Ca^2+^ stores. (**A**) In un-transfected HEK293T cells, ATP induced Ca^2+^ release through IP3-receptors on the ER, and GPN induced lysosomal Ca^2+^ release. (**B**) A 2-min application of TPEN, a membrane-permeable chelator of luminal ER Ca^2+^, attenuated Ca^2+^ release from IP3-receptors stimulated by ATP in HEK293T cells. (**C**) A 2-min TPEN application did not significantly reduce GPN-induced lysosomal Ca^2+^ release in HEK293T cells. (**D**) Long-term TPEN treatment (20 min) abolished ER Ca^2+^ release upon ATP stimulation and GPN-induced lysosomal Ca^2+^ release in HEK293T cells loaded with Fura-2. (**E**) In HEK293T cells transfected with the IP3R-ligand binding domain with ER targeting sequence (IP3R-LBD-ER), the responses to ATP and GPN were reduced compared to un-transfected cells on the same coverslip. (**F**) Caffeine stimulates Ca^2+^ release from ryanodine receptors and ATP stimulates Ca^2+^ release from IP3Rs in HEK-GCaMP3-ML1 cells loaded with Fura-2. Panels **A**–**C** and **E** show the average response of 30–40 cells from one representative experiment. **DOI:** http://dx.doi.org/10.7554/eLife.15887.011

[Ca^2+^]_ER_, but not cytosolic Ca^2+^, can be chelated by *N*,*N,N',N'-Tetrakis* (2-pyridylmethyl) ethylenediamine (TPEN), a membrane-permeable metal chelator with a low affinity for Ca^2+^ (Hofer et al., 1998). Although TPEN may also enter the lysosomal lumen, the much reduced (>100 fold less) Ca^2+^-binding affinity in the acidic pH (pH_LY_ = 4.6) suggests that chelation of lysosomal Ca^2+^ would be minimal. Acute application of TPEN completely blocked lysosomal Ca^2+^ refilling (Figure 2F,G). A short application of TPEN also blocked ER Ca^2+^ release stimulated by the endogenous P2Y receptor agonist ATP (Figure 2—figure supplement 2B compared to Figure 2—figure supplement 2A), but not the lysosomal Fura-2 Ca^2+^ response stimulated by GPN (Figure 2—figure supplement 2C compared to Figure 2—figure supplement 2A). These findings suggest that chelation of ER Ca^2+^ stores using TPEN had no direct effect on the naïve Ca^2+^ store in lysosomes, but specifically and potently affected lysosomal Ca^2+^ refilling.

The ER Ca^2+^store can also be genetically and chronically reduced without raising intracellular Ca^2+^ levels by transfecting cells with the IP3R ligand-binding domain with an ER targeting sequence (IP3R-LBD-ER) (Várnai et al., 2005). As expected, IP3R-LBD-ER expression decreased ATP-induced IP3R-mediated Ca^2+^ release (Figure 2—figure supplement 2E). Interestingly, it also reduced the GPN induced lysosomal Ca^2+^ release in HEK293T cells (Figure 2—figure supplement 2E). Furthermore, in HEK-GCaMP3-ML1 cells transfected with IP3R-LBD-ER, lysosomal Ca^2+^ release was significantly reduced when compared to un-transfected cells on the same coverslip (Figure 2H,I). Collectively, these findings suggest that the ER, the major Ca^2+^ store in the cell, is essential for refilling and the ongoing maintenance of lysosomal Ca^2+^ stores, but not required for the naïve Ca^2+^ release from lysosomes.

A functional interaction between ER and lysosome Ca^2+^ stores was previously suggested (Haller et al., 1996a; 1996b), but these results have been largely ignored, presumably due to the lack of specific tools required for definitive interpretation. Recent findings have shown that as endosomes mature, they increase their contact with the ER (Friedman et al., 2013). Interestingly, the Ca^2+^ released from SERCA inhibition on the ER was detected on our GCaMP3-ML1 probe (Figure 2B,C, Figure 2—figure supplement 1D,F), likely due to close membrane contact between the ER and lysosomes (Eden, 2016). Similar detection of ER Ca^2+^ release by a genetically-encoded, lysosomally-targeted chameleon Ca^2+^ sensor utilizing lysosome membrane protein Lamp1 has also been reported (McCue et al., 2013). Using time-lapse confocal imaging, we found that the majority of lysosomes, marked by Lamp1-mCherry, move and traffic together with ER tubules, labeled with CFP-ER (Figure 2J,K). Thus, the ER could be the direct source of Ca^2+^ to lysosomes by forming nanojunctions with them (Eden, 2016).

### IP3-receptors, not ryanodine receptors, on the ER are required for Ca^2+^ refilling of lysosomes

**Figure 3.**
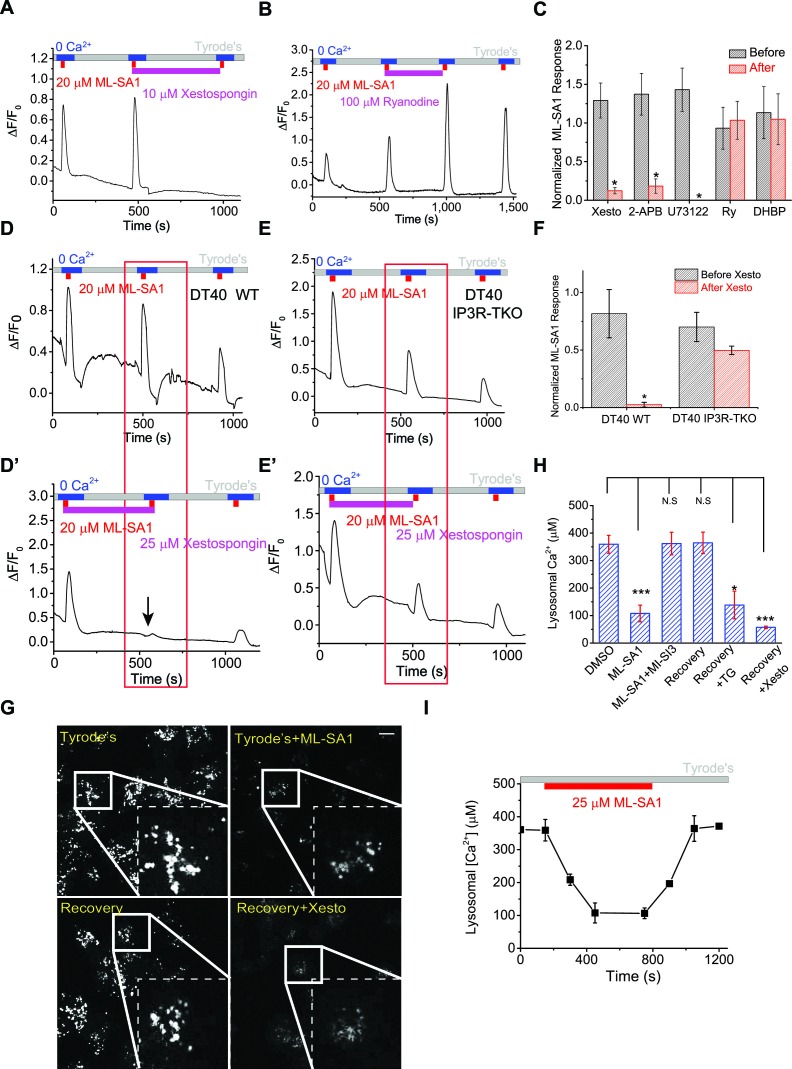
IP3-receptors on the ER are required for lysosomal Ca^2+^ store refilling. (**A**) The IP3-receptor (IP3R) antagonist Xestospongin-C (Xesto, 10 μM) prevented Ca^2+^ refilling of lysosomes in HEK-GCaMP3-ML1 cells (p=0.007). Note that Xesto was co-applied with ML-SA1. (**B**) Ryanodine (100 μM), which blocks Ryanodine receptors at high concentrations, did not block Ca^2+^ refilling to lysosomes. Note that Ryanodine was co-applied with ML-SA1. (**C**) Quantification of the responses to ML-SA1 in HEK-GCaMP3-ML1 cells after treatment with Xesto, 2-APB (Figure 3—figure supplement 1K), U73122 (Figure 3—figure supplement 1L,M), Ryanodine (Ry), and DHBP (Figure 3—figure supplement 2A) (Figure 3*—*source data 1).(**D**) DT40 WT cells transiently transfected with GCaMP3-ML1 show Ca^2+^ refilling. (**D’**) IP3R antagonist Xesto completely blocked Ca^2+^ refilling of lysosomes in DT40 WT cells. (**E**) DT40 IP3R triple KO (TKO) cells transiently transfected with GCaMP3-ML1 also show Ca^2+^ refilling. (**E’**) Xesto did not block Ca^2+^ refilling of lysosomes in IP3R-TKO cells. (**F**) Quantification of ML-SA1 responses with or without Xesto in WT and IP3R-TKO DT40 cells (Figure 3*—*source data 1). (**G**) Representative images showing the effects of Xesto on the recovery of ML-SA1-induced responses in HEK-ML1 stable cells loaded with OG-BAPTA-dextran. La^3+^ was used to block external Ca^2+^ influx that could be mediated by surface-expressed ML1 in the overexpression system (see Figure 1—figure supplement 2G). (**H**) The effects of TG and Xesto on intralysosomal Ca^2+^ contents measured by OG-BAPTA-dextran (Figure 3*—*source data 1). (**I**) The effects of ML-SA1 on [Ca^2+^]_Ly_ measured by OG-BAPTA-dextran. Panels **A**, **B**, **D**, **D’**, **E**, **E’**, **F**, **F’** and **H** are the average of 30–40 cells from one representative experiment. The data in panels **C**, **F** and **H** represent mean ± SEM from at five independent experiments. The scale bar in panel G = 10 μm. **DOI:** http://dx.doi.org/10.7554/eLife.15887.012 10.7554/eLife.15887.013Figure 3—source data 1.Normalized ML-SA1 responses or lysosomal Ca^2+^ contents under pharmacological (**C**, **H**) or genetic manipulations (**F**) (Figure 3C, F, H). The effects of Xesto on GPN responses in GCaMP3-ML1 (**G**) and MEF cells (**J**) (Figure 3—figure supplement 1G, J). ML-SA1 responses in GCaMP3-ML1-transfected WT and IP3R-TKO DT40 cells (Figure 3—figure supplement 2B). The effects of Xesto treatment on lysosomal Ca^2+^ changes induced by ML-SA1 (Figure 3—figure supplement 3E, G).**DOI:** http://dx.doi.org/10.7554/eLife.15887.013

Ca^2+^ release from the ER is mediated primarily by two Ca^2+^ release channels, IP3Rs and ryanodine receptors (RYRs), both of which are expressed in HEK cells (Querfurth et al., 1998; Jurkovicova et al., 2008) (see also Figure 2—figure supplement 2F). Since IP3Rs are responsible for Ca^2+^ transfer to mitochondria (Hayashi et al., 2009), we examined whether IP3Rs on the ER were responsible for Ca^2+^ refilling of the lysosome. Notably, Ca^2+^ refilling of the non-naïve lysosome Ca^2+^ store was completely blocked by Xestospongin-C (Xesto; Figure 3A,C), a relatively specific IP3R blocker (Peppiatt et al., 2003) (Figure 3—figure supplement 1A–E). In addition, in the GPN & Fura-2 assay that provides a measurement of lysosomal Ca^2+^ release independent of ML1, blocking IP3 receptor by Xesto profoundly attenuated lysosomal Ca^2+^ refilling in both HEK-GCaMP3-ML1 cells and non-transfected mouse embryonic fibroblasts (MEF) cells (Figure 1D; Figure 3—figure supplement 1F–J). Acute application of Xesto after allowing lysosomal Ca^2+^ stores to refill for 5 min (hence stores are completely refilled and functionally equivalent to 'naïve' ones) also slowly (up to 10 min) reduced lysosomal Ca^2+^ release, suggesting that constitutive lysosomal Ca^2+^ release under resting conditions may gradually deplete lysosome Ca^2+^ stores if refilling is prevented (Figure 3—figure supplement 1B–E). Consistent with this hypothesis, long-term (20 min) treatment with the aforementioned ER Ca^2+^ manipulators including TG and TPEN almost completely abolished lysosomal Ca^2+^ release (Figure 2—figure supplement 2D), further supporting the interpretation that ongoing constitutive Ca^2+^ release and refilling requires ER Ca^2+^.

**Figure 3—figure supplement 1.**
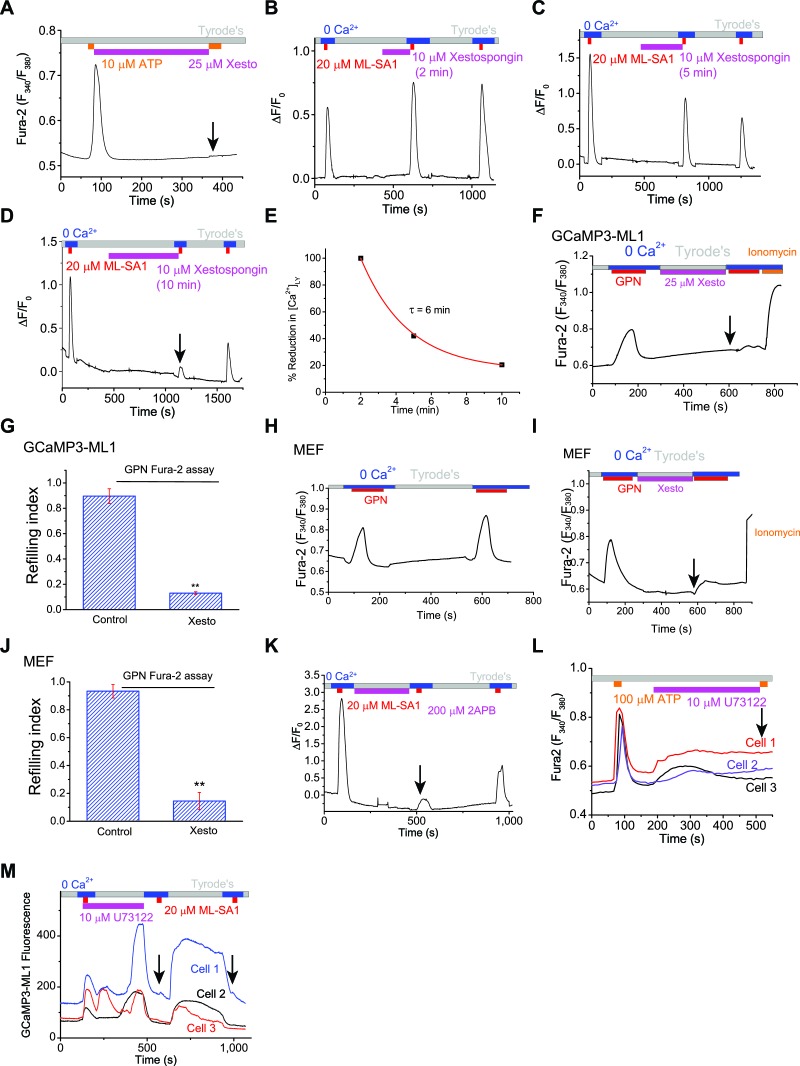
ER IP3-Receptors regulate Ca^2+^ refilling of lysosomes. (**A**) Xesto application (5 min) blocked ER Ca^2+^ release in HEK293T cells loaded with Fura-2. (**B**) After 5 min of refilling, which is expected to fully refill the lysosomal Ca^2+^ stores, acute treatment of Xesto (10 μM) for 2 min did not significantly reduce lysosomal Ca^2+^ release. Lysosomal Ca^2+^ release was induced by ML-SA1 in HEK-GCaMP3-ML1 cells. (**C**) After 5 min of refilling of lysosomal Ca^2+^ stores, subsequent acute treatment of Xesto (10 μM) for 5 min slightly reduced lysosomal Ca^2+^ release. (**D**) After 5 min of refilling of lysosomal Ca^2+^ stores, acute treatment of Xesto (10 μM) for 10 min abolished lysosomal Ca^2+^ release. (**E**) Time-dependent depletion of lysosomal Ca^2+^ stores by pharmacological inhibition of IP3-receptors. (**F**, **G**) In contrast to control (Figure 1C), application of Xesto dramatically reduced Fura-2 responses to GPN in GCaMP3-ML1 cells loaded with Fura-2 (Figure 3*—* source data 1). (**H**) Fura-2 Ca^2+^ imaging of GPN responses in MEF cells. (**I**) Xesto application dramatically reduced the second response to GPN in MEF cells. (**J**) Average effects of Xesto on lysosomal Ca^2+^ refilling in MEF cells (Figure 3*—*source data 1). (**K**) IP3R antagonist 2-APB (200 μM) blocked lysosomal Ca^2+^ refilling (p=0.013; also see Figure 3C). (**L**) PLC inhibitor U73122 (10 μM) blocked Ca^2+^ release from IP3Rs stimulated by ATP. (**M**) U73122 treatment abolished Ca^2+^ refilling of lysosomes (p=0.0070). Panels **A**–**D**, **F**, **H**, **I**, **K**, **L** and **M** show the average response of 30–40 cells from one representative experiment. **DOI:** http://dx.doi.org/10.7554/eLife.15887.014

**Figure 3—figure supplement 2.**
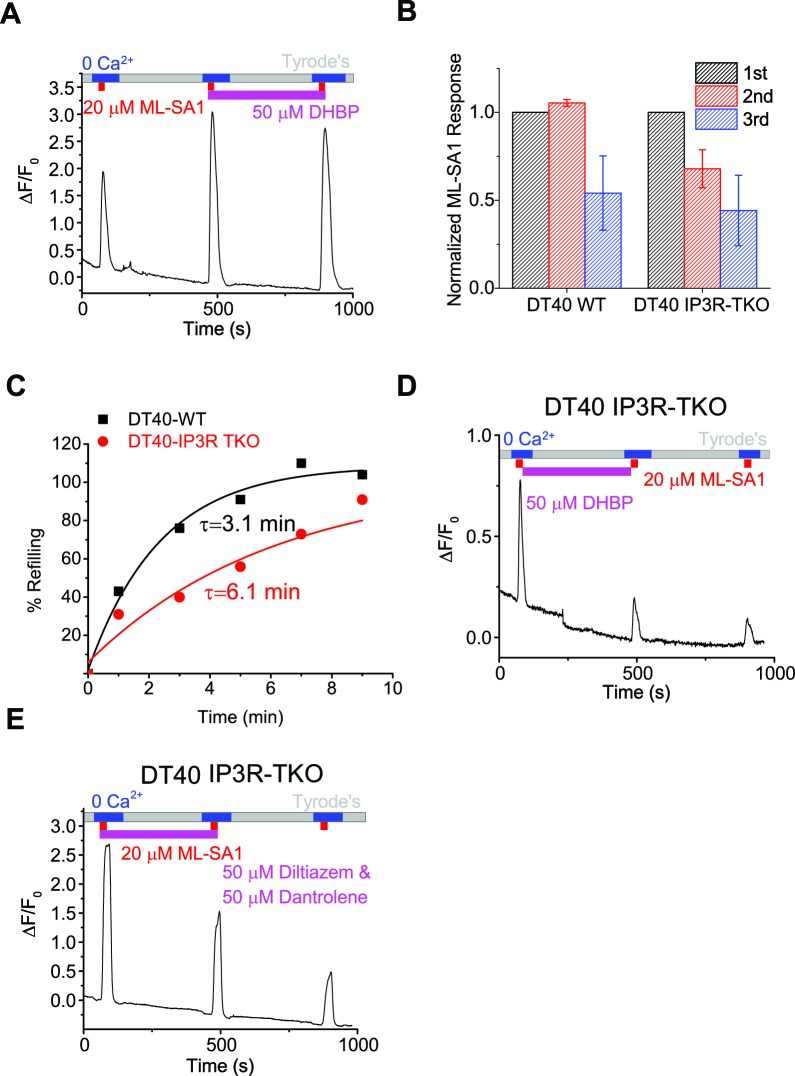
Lysosomal Ca2+ refilling is compromised in IP3R TKO DT40 cells. (**A**) Ryanodine receptor blocker DHBP (50 μM) did not block Ca^2+^ refilling of lysosomes. (**B**) Quantification of the 1st, 2nd and 3rd ML-SA1 responses in GCaMP3-ML1-transfected WT and IP3R-TKO DT40 cells (Figure 3*—*source data 1). (**C**) Time-dependence of lysosomal Ca^2+^ store refilling in WT and IP3R TKO DT40 cells. (**D**) GCaMP3-ML1-transfected IP3R-TKO DT40 cells still showed refilling after 5 min of DHBP application to block RYRs. (**E**) RYR inhibitors Diltiazem (50 µM) and Dantrolene (50 µM) did not block lysosomal Ca^2+^ refilling in GCaMP3-ML1-transfected IP3R-TKO DT40 cells. Panels **A**, **D** and **E** show the average response of 30–40 cells from one representative experiment. **DOI:** http://dx.doi.org/10.7554/eLife.15887.015

**Figure 3—figure supplement 3.**
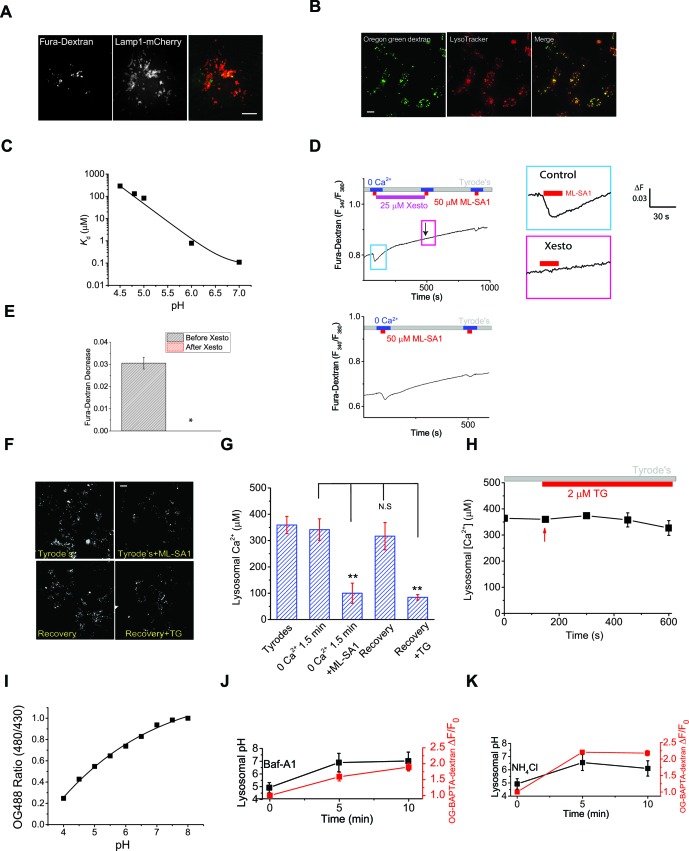
Measuring lysosomal Ca^2+^ release with lysosome-targeted luminal Ca^2+^ indicators. (**A**) Fura-Dextran was pulse/chased into HEK293T cells transfected with Lamp1-mCherry. Fura-Dextran dyes were co-localized well with Lamp1-mCherry after 12 hr pulse and 4 hr chase, although not all lysosomes were loaded with the dye, evidenced by many Lamp1-mCherry vesicles without Fura-Dextran co-localization. Scale bar = 5 μm. (**B**) OG-BAPTA-dextran displayed better loading to lyssosomes and a high level of co-localization with LysoTracker. Scale bar =10 μm. (**C**) pH-dependence of the measured *K*_d_ values for OG-BAPTA-dextran. (**D**) Compared with the control, Xesto (25 μM) treatment for 5 min prevented Ca^2+^ refilling to lysosomes measured with Fura-Dextran, a lysosome-targeted luminal Ca^2+^ indicator. Right panels show the zoom-in images of ML-SA1-induced responses before and after Xesto treatment. (**E**) Quantification of ML-SA1 responses with or without Xesto in cells loaded with Fura-Dextran. Xesto significantly (p=0.026) blocked refilling as shown by no response to ML-SA1 after Xesto application during refilling (n=3; Figure 3*—* source data 1). (**F**) Representative images showing the effect of TG treatment on the recovery of ML-SA1-induced responses in OG-BAPTA-dextran loaded HEK-ML1 stable cells. (**G**) Average effects of TG on lysosomal Ca^2+^ refilling in OG-BAPTA-dextran loaded HEK-ML1 cells. Lysosomal Ca^2+^ release was induced by ML-SA1 in zero Ca^2+^ external solution (Figure 3*—*source data 1). (**H**) The effects of TG treatment on intra-lysosomal Ca^2+^ levels, measured by OG-BAPTA-dextran imaging. (**I**) A calibration curve for the pH-sensitive dye OG-488-dextran. (**J**, **K**) Baf-A1 (5 μM; **J**) and NH_4_Cl (10 mM; **K**) induced changes in both lysosomal pH and OG-BAPTA-dextran fluorescence. **DOI:** http://dx.doi.org/10.7554/eLife.15887.016

2-APB, a non-specific IP3R antagonist (Peppiatt et al., 2003), also blocked Ca^2+^ refilling (Figure 3C, Figure 3—figure supplement 1K). U73122 is a PLC inhibitor that blocks the constitutive production of IP3 (Cárdenas et al., 2010) and prevents ATP-induced IP3R-mediated Ca^2+^ release (Figure 3—figure supplement 1L). U73122 also completely prevented Ca^2+^ refilling of lysosomes (Figure 3C, Figure 3—figure supplement 1M), suggesting that basal production of IP3 is essential for Ca^2+^ refilling of lysosomes. In contrast, blocking the RyRs with high (>10 μM) concentrations of ryanodine (Figure 3B,C), or with the receptor antagonist 1,1′-diheptyl-4,4′-bipyridinium (DHBP) (Berridge, 2012) (Figure 3C, Figure 3—figure supplement 2A), did not affect Ca^2+^ refilling. Notably, co-application of RYR and IP3R blockers with the second ML-SA1 response did not change the amplitude of the response (Figure 3A,B). Together, these findings demonstrate that IP3Rs on the ER are specifically required for lysosomal Ca^2+^ refilling, but not for Ca^2+^ release from naïve stores or completely refilled stores.

In contrast with the pharmacological analyses described above, lysosomal store refilling occurred in both WT and IP3R triple KO (TKO) DT40 chicken B cells (Várnai et al., 2005; Cárdenas et al., 2010) that were transfected with GCaMP3-ML1 (Figure 3D–F, Figure 3—figure supplement 2B). However, unlike WT DT40 cells, in which the IP3R-specific antagonist Xesto completely blocked Ca^2+^ refilling (Figure 3D’, F), Xesto had no obvious blocking effect in IP3R-TKO cells (Figure 3E’, F). In addition, the kinetics of lysosomal refilling was markedly delayed in IP3R TKO cells compared with WT cells (Figure 3—figure supplement 2C). These results are consistent with the notion that in normal conditions, IP3Rs are the sole source of Ca^2+^ refilling of lysosomes. When IP3Rs are genetically deleted, however, IP3R-independent mechanisms contribute to lysosomal Ca^2+^ refilling, possibly as a consequence of genetic compensation. Refilling in IP3R-TKO DT40 cells was not blocked by RYR inhibitors (Figure 3—figure supplement 2D,E).

### Studying lysosomal Ca^2+^ refilling using intra-lysosomal Ca^2+^ dyes

As an additional assay to directly 'monitor' intralysosomal Ca^2+^, we employed two intraluminal Ca^2+^ indicators Fura-Dextran and Oregon 488 BAPTA-1 dextran (OG-BAPTA-dextran) (Morgan et al., 2015). After being pulse/chased into ML1-mCherry-transfected HEK293T cells or HEK-ML1 stable cells, the dyes enter the lysosome lumen (Figure 3—figure supplement 3A,B) after endocytosis (Christensen et al., 2002). Due to their pH sensitivities, these dyes can detect intra-lysosomal Ca^2+^ ([Ca^2+^]_LY_) changes, but preferentially only when the intra-lysosomal pH (pH_L_) remains constant below pH 5.0 (Morgan et al., 2015) (see Figure 3—figure supplement 3C). In the Fura-Dextran-loaded ML1-mCherry-transfected HEK-293T cells, ML-SA1 application induced Ca^2+^ release from the lysosome lumen (Figure 3—figure supplement 3D). As we found in our GCaMP3-ML1 assay, Xesto abolished the ML-SA1-induced decrease in [Ca^2+^]_LY_(Figure 3—figure supplement 3D,E). Likewise, in HEK-ML1 stable cells loaded with OG-BAPTA-dextran, which had a much higher efficiency in loading to the lysosome (Figure 3—figure supplement 3B), TG or Xesto treatment profoundly reduced lysosomal Ca^2+^ refilling (Figure 3G—I; Figure 3—figure supplement 3F,G). Note that LysoTracker staining was not significantly reduced by ML-SA1, TG, or Xesto, suggesting that the signals were primarily mediated by changes of intralysosomal Ca^2+^, not intralysosomal pH. In contrast, treatment of cells with Baf-A1 or NH_4_Cl markedly increased lysosomal pH from 4.8 to 7.0 (Figure 3—figure supplement 3J,K). Such large pH elevations may cause dramatic changes in both *K*_d_ of OG-BAPTA-dextran (see Figure 3—figure supplement 3C) and luminal Ca^2+^ buffering capability (Morgan et al., 2015; Dickson et al., 2012), preventing accurate determinations of [Ca^2+^]_LY_ under these pH manipulations. Taken together, these results are consistent with the conclusions that were drawn based on the aforementioned ML-SA1 & GCaMP3-ML1 assay and the GPN & Fura-2 assay.

### Inhibition of ER IP3R channels and Ca^2+^ release causes lysosomal dysfunction and a LSD-like phenotype

**Figure 4.**
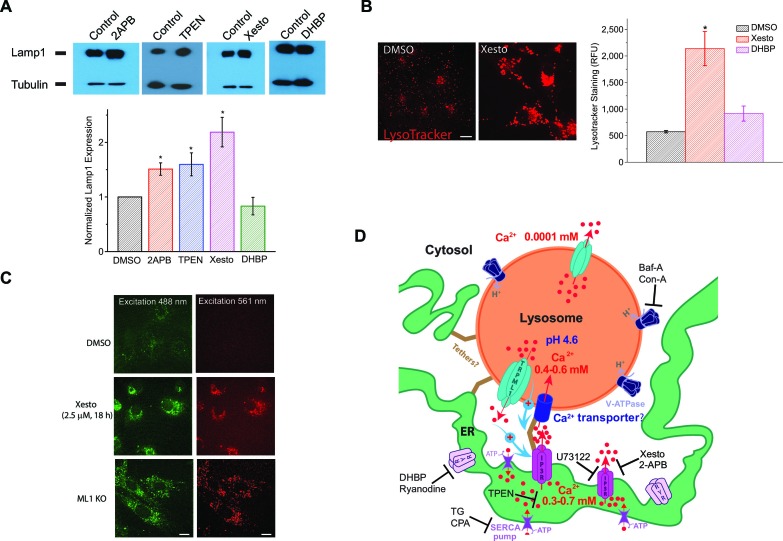
Blocking ER IP3-receptors Ca^2+^ channels refill lysosome Ca^2+^ stores to prevent lysosomal dysfunction. (**A**) Upper panels: Western blotting analyses of Lamp1 in HEK293T cells treated with 2-APB (50 μM), TPEN (0.1 μM), Xesto (10 μM), and DHBP (5 μM) compared to DMSO for 24 hr (n=4 separate experiments for each condition). Lower panel: treating HEK293T cells with 2-APB (p=0.05) and Xesto (p=0.013), as well as TPEN (p=0.02), significantly increased Lamp1 expression. DHBP did not significantly change Lamp1 expression (p=0.23) (Figure 4*—*source data 1). (**B**) The effects of Xesto (10 μM, 18 hr; p=0.0001) and DHBP (50 μM, 18 hr; p=0.063) treatment compared to DMSO on the lysosomal compartments detected by LysoTracker staining in HEK293T cells (average of 20–30 cells in each of 3 experiments; Figure 4*—*source data 1). Scale bar = 15 μm. (**C**) The effect of Xesto (10 μM, 18 h) treatment on accumulation of the autofluorescent lipofuscin materials in non-transfected HEK293T cells. Autofluorescence was observed in a wide spectrum but shown at two excitation wavelengths (488 and 561 nm). ML1 KO MEFs are shown for comparison. Scale bar = 15 μm. (**D**) A proposed model of Ca^2+^ transfer from the ER to lysosomes. The ER is a Ca^2+^ store with [Ca^2+^]_ER_ ~ 0.3–0.7 mM; lysosomes are acidic (pH_Ly_ ~ 4.6) Ca^2+^ stores ([Ca^2+^]_Ly_ ~ 0.5 mM). IP3Rs on the ER release Ca^2+^ to produce a local high Ca^2+^ concentration, from which an unknown low-affinity Ca^2+^ transport mechanism refills Ca^2+^ to a lysosome. Unidentified tether proteins may link the ER membrane proteins directly with the lysosomal membrane proteins to maintain contact sites of 20–30 nm for purposes of Ca^2+^ exchange. Ca^2+^ released from lysosomes or a reduction/depletion in [Ca^2+^]_Ly_ may, through unidentified mechanisms, 'promote' or 'stabilize' ER-lysosome interaction (Phillips and Voeltz, 2016; Eden, 2016). At the functional ER-lysosome contact sites, Ca^2+^ can be transferred from the ER to lysosomes through a passive Ca^2+^ transporter or channel based on the large chemical gradient of Ca^2+^ that is created when lysosome stores are depleted. Baf-A and Con-A are specific V-ATPase inhibitors; Xesto and 2APB are IP3R blockers; U73122 is a PLC inhibitor that blocks the constitutive production of IP3; DHBP and Ryanodine (>10 μM) are specific RyR blockers; TG and CPA are SERCA pump inhibitors; and TPEN is a luminal Ca^2+^ chelator. **DOI:** http://dx.doi.org/10.7554/eLife.15887.017 10.7554/eLife.15887.018Figure 4—source data 1.Source data of Figure 4A,B: Quantifications of Lamp-1 protein levels (**A**) or LysoTracker staining (**B**) under different experimental conditions and manipulations.**DOI:** http://dx.doi.org/10.7554/eLife.15887.018

Lysosomal Ca^2+^ is important to lysosomal function and membrane trafficking (Kiselyov et al., 2010; Shen et al., 2012; Lloyd-Evans et al., 2008). Lysosomal dysfunction is commonly associated with a compensatory increase of lysosome biogenesis, manifested as increased expression of essential lysosomal genes (Settembre et al., 2013). For example, the expression of Lamp1, a lysosomal marker, is elevated in most LSDs (Meikle et al., 1997). Lamp1 expression was significantly elevated in cells treated with low concentrations of IP3R blockers 2-APB and Xesto, as well as the ER Ca^2+^ chelator TPEN, but not in the cells treated with the RyR blocker DHBP (Figure 4A). Consistently, LysoTracker staining was significantly increased in cells treated with Xesto, but not 1,1'-diheptyl-4,4'-bipyridinium dibromide (DHBP; Figure 4B). Lysosomal dysfunction is also often associated with lysosomal enlargement and accumulation of various incompletely digested biomaterials (Shen et al., 2012; Dong et al., 2008). Notably, in cells that were treated with Xesto, but not with the vehicle control DMSO, lysosomal compartments were enlarged, and non-degradable, autofluorescent lipofuscin-like materials accumulated in puncta structures (Figure 4C), reminiscent of cells with defective lysosomal Ca^2+^ release (ML1 KO cells; Dong et al., 2008) (Figure 4C). By showing that inhibiting IP3R-mediated Ca^2+^ release from the ER results in a lysosome storage phenotype in the cell, these findings suggest that lysosome Ca^2+^ store refilling from IP3Rs ion the ER has important consequences for lysosome function and cellular health.

## Discussion

Using pharmacological and genetic approaches to manipulate ER Ca^2+^ levels and Ca^2+^ release and three different assays to directly measure lysosome Ca^2+^ release, we show that under normal conditions lysosome Ca^2+^ stores are refilled from the ER Ca^2+^ store through IP3 receptors independent of lysosome pH (see Figure 4D). Our findings are in contrast to several studies in the literature that suggest that inhibition of the V-ATPase is sufficient to deplete lysosome Ca^2+^ stores. Previous conclusions suggesting the importance of H^+^ gradient in regulating lysosome Ca^2+^ stores would therefore implicate the existence of an H^+^-dependent Ca^2+^ transporter in lysosomal membranes that can operate at the extremely low cytosolic free Ca^2+^ level (100 nM), representing a high affinity uptake system. Our work, however, suggest that a low affinity uptake mechanism is more likely. Hence either a low affinity Ca^2+^ transporter or rectifying Ca^2+^ channel might suffice. A putative VDAC-like channel in the lysosome, resembling mitochondrial VDAC channels (van der Kant and Neefjes, 2014), may interact directly with IP3Rs to receive Ca^2+^ from the ER. Importantly, it has been previously suggested that Ca^2+^ uptake into isolated lysosomes is mediated by a low-affinity (mM range) Ca^2+^ transporter (Lemons and Thoene, 1991).

The lysosomal pH gradient is thought to be essential for the maintenance of high free [Ca^2+^]_Ly_ (Calcraft et al., 2009; Christensen et al., 2002; Dickson et al., 2012; Lloyd-Evans et al., 2008; Shen et al., 2012). However, in addition to triggering lysosomal Ca^2+^ release, as proposed by Christensen et al. (Christensen et al., 2002), lysosomal pH elevation is also known to affect [Ca^2+^]_Ly_ or its measurement via several other mechanisms. Whereas the total [Ca^2+^]_Ly_ is reported to be in the low mM range (5–10 mM), free [Ca^2+^]_Ly_ is generally agreed to be in the high μM range (100–500 μM) (Morgan et al., 2015). Therefore, the lysosome lumen must contain substantial Ca^2+^ buffers (Morgan et al., 2015). Ca^2+^ buffers in acidic compartments and the ER are known to bind Ca^2+^ much better at neutral pH (Dickson et al., 2012). Hence increasing pH_L_ from 4.8 to 7.0 may effectively reduce free [Ca^2+^]_Ly_ without necessarily triggering lysosomal Ca^2+^ release and affecting total [Ca^2+^]_Ly_. Consistent with such an interpretation, a compelling study recently demonstrated that in secretory granules and the ER, increasing luminal pH changed the Ca^2+^ buffering capacity of both Ca^2+^ containing compartments and reduced free [Ca^2+^], while causing a minimal (20 nM) increase in cytosolic Ca^2+^ (Dickson et al., 2012). Additionally, lysosomal pH may act on luminal Ca^2+^ dyes by affecting their chromophore fluorescence and Ca^2+^-binding affinity (*K*_d_) (Morgan et al., 2015). Because *K*_d_ drops more than 1,000 foldtimes when pH_L_ is increased from 4.8 to 7.0, accurate calibration is currently not possible. Furthermore, prolonged lysosomal pH manipulations may also indirectly affect lysosomal Ca^2+^ homeostasis, for instance, via membrane fusion and fission between compartments containing different amounts of Ca^2+^, H^+^, and their buffers. Finally, although elevating lysosomal pH may trigger lysosomal Ca^2+^ release, the accompanying increase in cytoplasmic Ca^2+^ was rather small (20–40 nM) (Dickson et al., 2012; Christensen et al., 2002). Moreover, the instantaneous changes (following pH increase and decrease) of Ca^2+^ probe fluorescence (Dickson et al., 2012; Christensen et al., 2002) are inconsistent with the slow rates of Ca^2+^ leak and re-uptake demonstrated in the current study.

The persistence of a GPN signal even after intracellular Ca^2+^ chelation is important for understanding the limits of this lysosome-specific pharmacological tool. GPN can certainly be used in conjunction with other tools to examine lysosome specificity, but caution is necessary with its use for Ca^2+^ store measurement, as a component of the signal observed in Fura-2 loaded cells, although small, is a result of the membrane permeabilization that causes a decrease in cytosolic pH. Similarly, reagents like Baf-A and NAADP that are used to mobilize lysosomal Ca^2+^ also release H^+^ into the cytosol (Morgan and Galione, 2007; Appelqvist et al., 2012; Scott and Gruenberg, 2011; Yoshimori et al., 1991), which could have been misinterpreted as a Ca^2+^ signal in previous studies (Morgan et al., 2011). pH may affect cytosolic Ca^2+^ indicators through the chromophore fluorescence, Ca^2+^-binding affinity, or Ca^2+^-dependent conformational changes (e.g., in the case of GCaMP) (Morgan et al., 2015). Therefore, if experimental conditions are not optimized, the presumed cytosolic Ca^2+^ signals may also reflect pH changes, or unidentified pH-mediated non-Ca^2+^ signals. We propose that BAPTA-AM control experiments be routinely conducted in any lysosomal Ca^2+^ measurement. It is possible that the 'pH contaminating effect' might have resulted in numerous misinterpretations of lysosome Ca^2+^ stores in the literature, particularly those examining the interactions between ER and lysosome Ca^2+^.

Based on our results in the current study, recent studies of ER-lysosome interactions (Phillips and Voeltz, 2016), and previous Ca^2+^ uptake studies on isolated lysosomes (Lemons and Thoene, 1991), we hypothesize that ER-refilling of lysosomal stores is a regulated, two-step process (see Figure 4D). First, lysosome store depletion may trigger establishment of ER-lysosome contacts (Phillips and Voeltz, 2016). Although lysosomes and ER are in close proximity under resting conditions, lysosome store depletion may 'stabilize' the ER-lysosome contact, and/or 'tether' and approximate both membranes (e.g., from 20–30 nm to 10 nm) (Phillips and Voeltz, 2016; Eden, 2016). Second, at the relatively stable, functional ER-lysosome contact sites, a passive Ca^2+^ transport process can occur from the ER to lysosomes, by utilizing the large Ca^2+^gradient created when lysosome stores are actively depleted. Up to 5 min may be required to complete the whole refilling process from 'initiation' through 'uptake'.

Our results not only provide an explanation for the reported sensitivity of the Ca^2+^ stores of acidic organelles to ER disrupting agents (Menteyne et al., 2006; Haller et al., 1996a), but are also consistent with the observations that lysosomes may buffer cytosolic Ca^2+^ released from the ER (López-Sanjurjo et al., 2013). The unexpected role of the ER in maintaining Ca^2+^ stores in lysosomes may help resolve the long-standing mystery of how impaired ER Ca^2+^ homeostasis is commonly seen in lysosomal storage diseases (LSDs) (Coen et al., 2012), and how manipulating ER Ca^2+^ reduces lysosome storage (Lloyd-Evans et al., 2008; Mu et al., 2008). In addition, our work reveals that, depending on the treatment conditions (acute *versus* prolonged treatment), many assumed-to-be ER-specific reagents may indirectly affect lysosome Ca^2+^ stores. This may impact the interpretations of a large body of literature on Ca^2+^ signaling. Although we demonstrated a central role of IP3Rs in lysosomal Ca^2+^ refilling, other ER Ca^2+^ channels may also participate under certain conditions, as seen in the IP3R TKO cells.

Accumulating evidence suggests that the ER forms membrane contact sites with other organelles, including plasma membrane, mitochondria (Cárdenas et al., 2010), endosomes (Alpy et al., 2013), and lysosomes (van der Kant and Neefjes, 2014). ER-endosome membrane contact, although currently difficult to study, was proposed to facilitate cholesterol transport from endosomes to the ER (Rocha et al., 2009; van der Kant and Neefjes, 2014; Drin et al., 2016). Given the established role of lysosomal Ca^2+^ release in cholesterol transport (Shen et al., 2012), lysosomal Ca^2+^ release may have a direct role in regulating ER-lysosome interaction (see Figure 4D). In ER-mitochondria contact sites, the tethering protein GRP-75 links IP3Rs with VDAC channels on mitochondria to regulate Ca^2+^ homeostasis and ATP production (Cárdenas et al., 2010). Similar unidentified tethers may also link IP3Rs with the putative lysosomal Ca^2+^ transporter for store refilling (see Figure 4D). The importance of lysosomal Ca^2+^ in regulating a variety of intracellular signaling pathways is becoming increasingly recognized (Medina et al., 2015). ER-lysosome interaction may serve as a hub for Ca^2+^ signaling to regulate cellular homeostasis through coordinating the primary anabolic and catabolic pathways in the cell. Studying the two-step lysosomal Ca^2+^ refilling process may prove important for future identification of the low-affinity Ca^2+^ uptake transporter/channel in the lysosome, and for studying the molecular mechanisms that regulate the functional ER-lysosome interaction.

## Materials and methods

### Molecular biology

Genetically-encoded Ca^2+^ indicator GCaMP3 was fused directly to the N-terminus of ML1 (GCaMP3-ML1) as described previously (Shen et al., 2012). The IP3R-LBD-ER construct (Várnai et al., 2005) was a kind gift from Dr. Thomas Balla (National Institute of Child Health and Human Development, NIH). The pECFP-ER plasmid was obtained from CLONTECH. Lamp1-mCherry was made by fusing mCherry with the C terminus of Lamp1.

### Western blotting

Standard Western blotting protocols were used. HEK293T cells were treated every 4 hr for 24 hr with IP3R antagonists 2-APB and Xestospongin-C, ER Ca^2+^ chelator TPEN, and RyR antagonist DHBP. Lamp1 antibody was from Developmental Studies Hybridoma Bank (Iowa).

### Mammalian cell culture

Immortalized cell lines (HEK293 and Cos-7) were purchased from ATCC and cultured following standard culture protocols. DT40-WT and IP3R-TKO cells were a generous gift from Dr. Darren Boehning (The University of Texas Health Sciences Center at Houston). Human fibroblasts were obtained from the Cornell Institute for Medical Research (NJ, USA). HEK 293 cells stably expressing GCaMP3-ML1 (HEK-GCaMP3-ML1 cells) were generated using the Flip-In T-Rex 293 cell line (Invitrogen). All these cells were neither authenticated nor tested for mycoplasma contamination. HEK293 cells are on the list of frequently misidentified or cross-contaminated cell lines. All cells were cultured in a 37°C incubator with 5% CO_2_. HEK293T cells, Tet-On HEK293 cells stably expressing GCaMP3-ML1 (HEK-GCaMP3-ML1 cells), Cos-7 cells, and human fibroblasts were cultured in DMEM F12 (Invitrogen) supplemented with 10% (vol/vol) FBS or Tet-free FBS. DT40 cells were kept in suspension in RPMI 1640 (Invitrogen) supplemented with 450 µL β-mercaptoethanol, 2 mM L-glutamine, 10% FBS, and 1% chicken serum (Várnai et al., 2005; Cárdenas et al., 2010). We noted that lysosomal Ca^2+^ store refilling was often compromised in high-passage or poorly-maintained cell cultures.

Human fibroblasts and DT40 cells were transiently transfected using the Invitrogen Neon electroporation kit (1200 V, 1 pulse, 30 s). HEK293T cells, HEK-GCaMP3-ML1 cells, and Cos-7 cells were transfected using Lipofectamine 2000 (Invitrogen). All cells were used for experiments 24–48 hr after transfection.

### Confocal imaging

Live imaging of cells was performed on a heated and humidified stage using a Spinning Disc Confocal Imaging System. The system includes an Olympus IX81 inverted microscope, a 100X Oil objective NA 1.49 (Olympus, UAPON100XOTIRF), a CSU-X1 scanner (Yokogawa), an iXon EM-CCD camera (Andor). MetaMorph Advanced Imaging acquisition software v.7.7.8.0 (Molecular Devices) was used to acquire and analyze all images. LysoTracker (50 nM; Invitrogen) was dissolved in culture medium and loaded into cells for 30 min before imaging. MitoTracker was dissolved in culture medium and loaded into cells for 15 min before imaging (25 nM). Coverslips were washed 3 times with Tyrode’s and imaged in Tyrode’s.

### GCaMP3-ML1 Ca^2+^ imaging

GCaMP3-ML1 expression was induced in Tet-On HEK-GCaMP3-ML1 cells 20-24h prior to experiments using 0.0 1µg/mL doxycycline. GCaMP3-ML1 fluorescence was monitored at an excitation wavelength of 470 nm (F_470_) using a EasyRatio Pro system (PTI). Cells were bathed in Tyrode’s solution containing 145 mM NaCl, 5 mM KCl, 2 mM CaCl_2_, 1 mM MgCl_2_, 10 mM Glucose, and 20 mM Hepes (pH 7.4). Lysosomal Ca^2+^ release was measured in a zero Ca^2+^ solution containing 145 mM NaCl, 5 mM KCl, 3 mM MgCl_2_, 10 mM glucose, 1 mM EGTA, and 20 mM HEPES (pH 7.4). Ca^2+^ concentration in the nominally free Ca^2+^ solution is estimated to be 1–10 μM. With 1 mM EGTA, the free Ca^2+^ concentration is estimated to be <10 nM based on the Maxchelator software (http://maxchelator.stanford.edu/). Experiments were carried out 0.5 to 6 hr after plating. Because baseline may drift during the entire course of the experiment (up to 20 min), we typically set F_0_ based on the value that is closest to the baseline.

### Fura-2 Ca^2+^ imaging

Cells were loaded with Fura-2 (3 µM) and Plurionic-F127 (3 µM) in the culture medium at 37°C for 60 min. Florescence was recorded using the EasyRatio Pro system (PTI) at two different wavelengths (340 and 380 nm) and the ratio (F_340_/F_380_) was used to calculate changes in intracellular [Ca^2+^]. All experiments were carried out 1.5 to 6 hr after plating.

### Oregon green 488 BAPTA-1 dextran imaging

Cells were loaded with Oregon Green 488 BAPTA-1 dextran (100 μg/ml) at 37°C in the culture medium for 4–12 hr, and then pulsed/chased for additional 4–16 hr. Fluorescence imaging was performed at 37°C. In vitro calcium-binding (*K*_d_) affinities of OG-BAPTA-dextran were determined using KCl-based solutions (140 mM KCl, X mM CaCl_2_, 1 mM MgCl_2_, 10 mM HEPES, 10 mM MES, 0 or 1 mM BAPTA) adjusted to different pH (pH 4.5, 5.0, 6.0, and 7.0). By varying the amount of added Ca^2+^ (X= 0- 10 mM), solutions with different pH and free [Ca^2+^] were made based on the Maxchelator software (http://maxchelator.stanford.edu/). OG-BAPTA-dextran (5 μg/ml) fluorescence for each solution was obtained to plot the calibration curve (Morgan et al., 2015; Dickson et al., 2012; Christensen et al., 2002). In cells that were pre-treated with ionomycin, nigericin, and valinomycin (Morgan et al., 2015; Dickson et al., 2012; Christensen et al., 2002), in vivo minimal and maximal Fluorescence (F_min_ and F_max_) were determined by perfusing the cells with 0 or 10 mM Ca^2+^ external solutions, respectively. Lysosomal [Ca^2+^] at different pH were determined using the following calibration equation: [Ca^2+^] = *K*_d_ × (F-F_min_)/ (F_max_-F).

### Lysosomal pH measurement

Cells were pulsed with OG-488-dextran for 6 hr, and chased for additional 12 hr (Johnson et al., 2016). Cells were then bathed in the external solutions (145 mM KCl, 5 mM glucose, 1 mM MgCl_2_, 1 mM CaCl_2_, 10 mM HEPES, 10 mM MES, adjusted to various pH values ranging from 4.0 to 8.0) that contained 10 μM nigericin and 10 μM monensin (Johnson et al., 2016). Images were captured using an EasyRatio Pro system. A pH standard curve was plotted based on the fluorescence ratios: F_480_/ F_430_.

### Cytosolic pH sensitivity of GCaMP3-ML1

GCaMP3-ML1-positive vacuoles were isolated from vacuolin-1-treated HEK-GCaMP3-ML1 cells, as described previously (Zhang et al., 2012). Briefly, cells were treated with 1 μM vacuolin-1 for up to 12 hr to increase the size of late endosomes and lysosomes (Cerny et al., 2004). Vacuoles were released into the dish by mechanical disruption of the cell membrane with a small glass electrode. After vacuoles were released into the dish, patch pipettes containing either a 'high-Ca^2+^' (10 mM) internal solution or a 'low-pH' solution (140 mM KCl, 1 mM EGTA, 20 mM MES, 10 mM Glucose, pH adjusted to 2.0) were placed close to 'puff' the vacuoles. Images were captured using a CCD camera connected to the fluorescence microscope.

### Reagents

All reagents were dissolved and stored in DMSO or water and then diluted in Tyrode’s and 0 Ca^2+^ solutions for experiments. 2-APB, ATP, Con-A, CPA, Doxycycline, DHBP, TG, TPEN were from Sigma; GPN and U73122 were from Santa Cruz; Ryanodine was from Abcam; LysoTracker, Fura-2, Mitotracker, Plurionic F-127, and Fura-Dextran were from Invitrogen; Baf-A was from LC Laboratories; ML-SA1 was from Chembridge; and Xestospongin-C was from Cayman Chemical, AG Scientific, and Enzo; Oregon Green 488 BAPTA-1 dextran was from life technologies. ML-SI compounds were identified from a Ca^2+^-imaging-based highthroughput screening conducted at NIH/NCATS Chemical Genomics Center (NCGC;https://pubchem.ncbi.nlm.nih.gov/bioassay/624414#section=Top). ML-SI compounds are available upon request.

### Data analysis

Data are presented as mean ± SEM. All statistical analyses were conducted using GraphPad Prism. Paired t-tests were used to compare the average of three or more experiments between treated and untreated conditions. A value of p<0.05 was considered statistically significant. In the cases only individual traces were shown, the traces are representative from at least 30–40 cells, or from at least independent repeats.
